# Microbial communities associated with the camel tick, *Hyalomma dromedarii*: 16S rRNA gene-based analysis

**DOI:** 10.1038/s41598-020-74116-7

**Published:** 2020-10-12

**Authors:** Nighat Perveen, Sabir Bin Muzaffar, Ranjit Vijayan, Mohammad Ali Al-Deeb

**Affiliations:** Biology Department, United Arab Emirates University, P.O. Box 15551, Al-Ain, UAE

**Keywords:** Microbiology, Microbial communities, Microbiome

## Abstract

*Hyalomma dromedarii* is an important blood-feeding ectoparasite that affects the health of camels. We assessed the profile of bacterial communities associated with *H. dromedarii* collected from camels in the eastern part of the UAE in 2010 and 2019. A total of 100 partially engorged female ticks were taken from tick samples collected from camels (*n* = 100; 50/year) and subjected to DNA extraction and sequencing. The 16S rRNA gene was amplified from genomic DNA and sequenced using Illumina MiSeq platform to elucidate the bacterial communities. Principle Coordinates Analysis (PCoA) was conducted to determine patterns of diversity in bacterial communities. In 2010 and 2019, we obtained 899,574 and 781,452 read counts and these formed 371 and 191 operational taxonomic units (OTUs, clustered at 97% similarity), respectively. In both years, twenty-five bacterial families with high relative abundance were detected and the following were the most common: *Moraxellaceae*, *Enterobacteriaceae*, *Staphylococcaceae*, *Bacillaceae*, *Corynebacteriaceae*, *Flavobacteriaceae*, *Francisellaceae*, *Muribaculaceae*, *Neisseriaceae*, *and Pseudomonadaceae*. *Francisellaceae* and *Enterobacteriaceae* coexist in *H. dromedarii* and we suggest that they thrive under similar conditions and microbial interactions inside the host. Comparisons of diversity indicated that microbial communities differed in terms of richness and evenness between 2010 and 2019, with higher richness but lower evenness in communities in 2010. Principle coordinates analyses showed clear clusters separating microbial communities in 2010 and 2019. The differences in communities suggested that the repertoire of microbial communities have shifted. In particular, the significant increase in dominance of *Francisella* and the presence of bacterial families containing pathogenic genera shows that *H. dromedarii* poses a serious health risk to camels and people who interact with them. Thus, it may be wise to introduce active surveillance of key genera that constitute a health hazard in the livestock industry to protect livestock and people.

## Introduction

Pathogens and the diseases they cause are of high importance to human and animal health^1,2^. Microbial and parasitic infections are ubiquitous in animal and human populations, and healthy ecosystems are often rich in pathogenic organisms^2,3^. In recent years, long-term host–pathogen associations have been disrupted primarily due to extensive anthropogenic changes in the environment resulting in emerging and re-emerging infectious diseases^1,2^. Density of hosts, vectors and pathogens in a geographic area are key determinants of disease transmission^4^. Globally, arthropods, such as ticks act as vectors of many human and animal pathogens (including viruses, bacteria and protozoa), often mediating transfer of infections from one host species to another^5–9^. Farming of animals throughout the world has resulted in artificially enhanced domestic animal populations. This in turn has increased tick abundance and distribution, particularly in peri-urban livestock industries.

Ticks feed exclusively on the blood of their vertebrate hosts^10^ and simultaneously harbor a variety of endosymbiotic and pathogenic microbes^11,12^. The assortment of bacteria harbored and transmitted by ticks is diverse, representing a wide range of genera including *Anaplasma*, *Borrelia*, *Coxiella*, *Cowdria*, *Ehrlichia*, *Francisella*, and *Rickettsia*. These bacteria are adapted to undergo development in the tick vector for at least a portion of their lifecycle^11,13^. In terms of *Francisella*, it is known that it may exist either as pathogenic species or as intracellar endosymbionts (*Francisella*-like endosymbiont). Though humans are considered accidental hosts of ticks, the bacterial diseases such as rickettsial diseases transmitted by various arthropod vectors affect an estimated one billion people worldwide^14,15^. Aside from causing human and animal morbidity and mortality, ticks and tick-borne diseases are responsible for huge global production losses, amounting to US$ 14–19 billion per annum^16^.

Rapid development in the Middle East region over the last 30 years has resulted in a large urban population composed of multiple ethnicities^17^. There has been a concomitant development in the farming industry, some of which are situated on the outskirts of cities, with a rise in camel farming throughout the region to support an increasing demand on camel milk and meat. Currently, there are over 459,000 camel heads in the United Arab Emirates ^18^, and over 1.6 million camel heads in the Arabian Peninsula^19^. *Hyalomma* tick species pose major threats to camels and other livestock across Africa, Eastern Europe, Middle East and Western Asia^20–22^. *Hyalomma* species are medium-sized to large ticks that parasitize domestic and wild mammals and birds, and are abundant in semi-arid zones^6,21^. *H. dromedarii* is the most common tick species that infests camels causing tick-borne diseases in camels and humans^21,23–27^. However, there has been relatively few studies on the ecology and biology of *H. dromedarii* ticks^28,29^. Tick infestations are recognized as an increasing problem in the camel industry throughout the year^30^ in the Arabian Peninsula and although a wide variety of acaricides are used to control ticks, their efficacy is not well characterized.

Next generation sequencing (NGS) technology has revolutionized genomic research by providing opportunities to analyze substantial amounts of genetic data at reduced cost^31–34^. Increasingly, microbiome studies are utilizing newer NGS platforms, such as the Illumina MiSeq, which have been reported to be more cost effective and accurate^33,35^. The V4 hypervariable region is usually selected for work on the MiSeq as it provides sufficient information for taxonomic profiling of microbial communities and has demonstrated a lower error rate on the Illumina platform^36^. Metagenomic approaches allow the investigation of entire bacterial microbiota associated with their vectors allowing better assessment of the diversity of circulating microbes and the reservoir potential of vectors^37,38^. Relatively less is known about the bacterial community structure associated with ticks. There is an increasing number of studies that have utilized metagenomic analysis of viral and microbial communities associated with ticks^37–43^. The establishment of the microbial community may be determined by host specificity of the microbe, with certain bacterial genera dominating in certain tick host species^44^. The vast majority of the microbes appear to be intracellular endosymbiotic such as *Coxiella*-, *Francisella*-, *Rickettsia*- and *Arsenophonus*-like symbionts^45^ and some of these endosymbionts often form complex interactions with pathogenic microbes in their tick hosts^40,46^. There is emerging evidence that diversity of microbial communities changes due to environmental conditions including temperature^43^, suggesting seasonality of the microbiota, which could in turn be linked with seasonality of pathogen transmission. Interestingly, the presence of some endosymbionts, such as *Rickettsia bellii* in *Dermacentor andersoni* ticks, is often associated with lowered infection rates of pathogenic species such as *Anaplasma marginale*, suggesting that endosymbionts may even play an integral role in suppressing pathogen transmission^46^. Thus, characterization of microbial communities in ticks is of great importance to our understanding of pathogen transmission to animals or humans^43,46^.

Nonetheless, so far there are no published records on the microbial communities in *H. dromedarii* ticks in the UAE. Two studies, one from West Bank, Palestine^47^, and second from Saudi Arabia^48^ investigated microbial communities in *H. dromedarii*. The objectives of this study were to (i) characterize the microbiota associated with *H. dromedarii* ticks in the UAE and (ii) determine temporal patterns of richness and evenness in microbial communities in *H*. *dromedarii* ticks.

## Results

### Microbial diversity in 2010

We obtained 899,574 read counts (average 89,957 sequences per sample) and these formed 371 operational taxonomic units (OTUs, clustered at 97% similarity), belonging to 10 phyla, 24 classes, 107 families, and 202 genera from 2010. Taxonomic profiling of the bacteria sampled from *H. dromedarii* confirmed seven abundant phyla: *Proteobacteria*, *Bacteroidetes*, *Firmicutes*, *Actinobacteria*, *Cynobacteria*, *Verrucomicrobia* and *Planctomycetes*. The phylum *Proteobacteria* was the most abundant while *Planctomycetes* had the least abundance (Supplementary Table 1).

**Table 1:** Significant multiple regression models that were retained after backward selection.

$$\begin{aligned} {\text{Y}}_{Acinetobacter} & =\upbeta _{0} +\upbeta _{Corynebacterium} +\upbeta _{Escherichia} +\upbeta _{Francisella} +\upbeta _{Lysin ibacillus} \\ & \quad +\upbeta _{Moraxella} +\upbeta _{Pseudomonas} +\upbeta _{Psychrobacter} +\upvarepsilon \left( {{\text{F}}_{(7,12)} = 7.02,\,p = 0.0005} \right) \\ \end{aligned}$$
$$\begin{aligned} {\text{Y}}_{Escherichia} & =\upbeta _{0} +\upbeta _{Acinetobacter} +\upbeta _{Corynebacterium} +\upbeta _{Francisella} +\upbeta _{Ly\sin ibacillus} \\ & \quad +\upbeta _{Moraxella} +\upbeta _{Pseudomonas} +\upbeta _{Psychrobacter} +\upvarepsilon \left( {{\text{F}}_{(7,12)} = 17.26,p < 0.0001} \right) \\ \end{aligned}$$
$${\text{Y}}_{Francisella} =\upbeta _{0} +\upbeta _{Acinetobacter} +\upbeta _{Escherichia} +\upvarepsilon \left( {\text{F}}_{(2,17)} = 7.21,p < 0.005 \right)$$

Out of 24 bacterial classes, 16 classes were abundant namely *Gammaproteobacteria*, *Flavobacteriia*, *Actinobacteria*, *Bacilli*, *Tissierellia*, *Bacteroidia*, *Clostridia*, *Betaproteobacteria*, *Alphaproteobacteria*, *Sphingobacteriia*, *Negativicutes*, *Chitinophagia*, *Planctomycetia*, *Erysipelotrichia*, *Deltaproteobacteria* and *Verrucomicrobiae. Gammaproteobacteria* was recorded as the dominant class in all locations except two (Supplementary Table 2).

**Figure 1:**
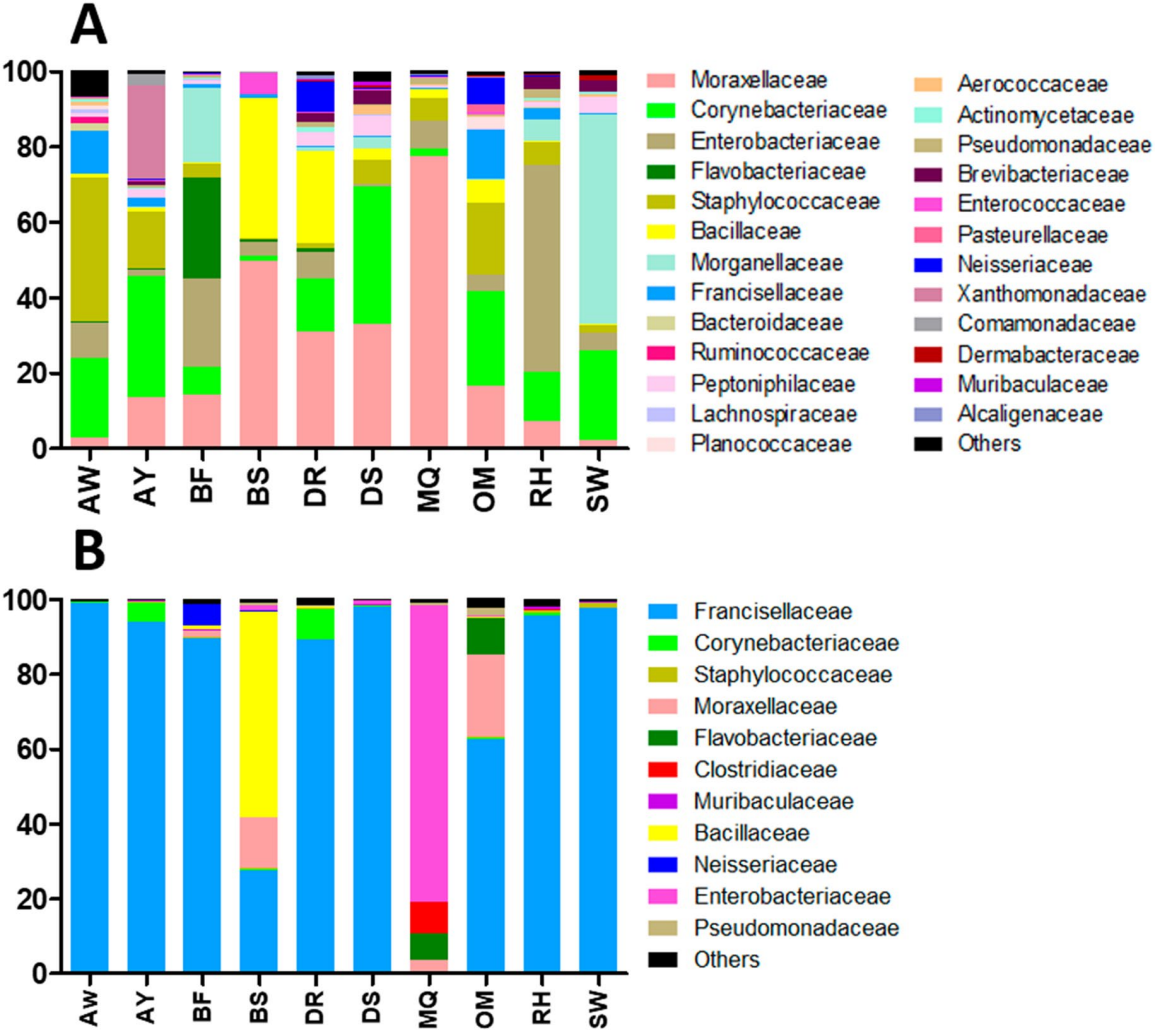
Microbial families detected in *H. dromedarii* adult ticks from ten locations in Al-Ain, UAE in 2010 (**A**) and 2019 (**B**). *AW* Al-Wagan, *AY* Al-Yahar, *BF* Bede’ Fares, *BS* Bede’Bent Suod, *DR* Dubai Road, *DS* Dwar Al-Shahenat, *MQ* Malaket, *OM* Omghafa, *RH* Remah, *SW* Swehan.

Taxonomic assignment showed that 25 bacterial families were more abundant (Supplementary Table 3). The ones with the highest relative abundance were: *Moraxellaceae* (77.52%), *Morganellaceae* (55.82%), *Enterobacteriaceae* (54.63%), *Staphylococcaceae* (38.1%), *Bacillaceae* (37.33%), *Corynebacteriaceae* (36.62%), *Flavobacteriaceae* (26.66%), *Xanthomonadaceae* (24.5%), *Francisellaceae* (11.4%) and *Neisseriaceae* (8%) in all of the sampled locations (Fig. 1A).

**Figure 2:**
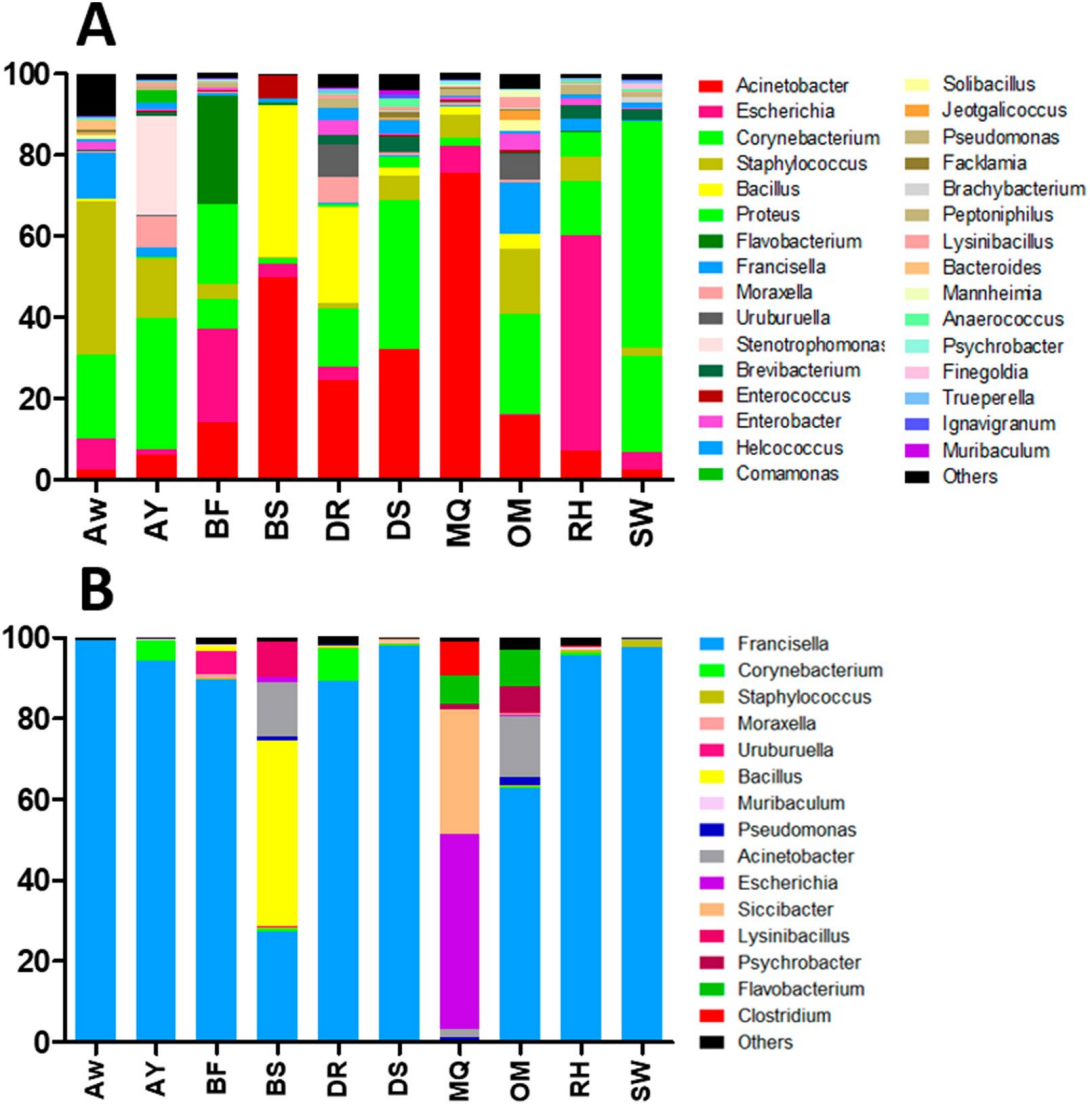
Microbial genera detected in *H. dromedarii* adult ticks from ten locations in Al-Ain, UAE in 2010 (**A**) and in 2019 (**B**). *AW* Al-Wagan, *AY* Al-Yahar, *BF* Bede’ Fares, *BS* Bede’Bent Suod, *DR* Dubai Road, *DS* Dwar Al-Shahenat, *MQ* Malaket, *OM* Omghafa, *RH* Remah, *SW* Swehan.

The relative abundance of genera was highly variable in the microbiome of *H. dromedarii* in all locations. *Acinetobacter* (75.66%) and *Corynebacterium* (36.62%) were the two most common genera with high relative abundance. *Proteus* had a relative abundance of 55.82% followed by *Escherichia* (53.13%) and *Staphylococcus* (37.68%). *Flavobacterium*, *Francisella*, *Moraxella*, *Uruburuella* and *Stenotrophomonas* occurred in moderately low relative abundance (6–25%). In addition, genera including *Enterobacter*, *Comamonas*, *Brevibacterium*, *Helcococcus*, *Facklamia*, *Anaerococcus*, *Ignavigranum* and *Muribaculum* were all low in terms of relative abundance (1–3.55%) (Fig. 2A; Supplementary Table 4).

### Microbial diversity in 2019

We obtained 781,452 sequences (average 78,145 sequences per sample) and these formed 191 unique OTUs belonging to 7 phyla, 18 classes, 65 families, and 109 genera from 2019. Profiling of the bacteria sampled from *H. dromedarii* species identified seven phyla: *Proteobacteria*, *Actinobacteria*, *Bacteroidetes*, *Firmicutes*, *Cynobacteria*, *Verrucomicrobia* and *Planctomycetes*. *Proteobacteria* was dominant in all locations except one location where *Firmicutes* had a high relative abundance (55.54%) as compared to *Proteobacteria* (43.80%) (Supplementary Table 5).

Taxonomic profiling revealed 14 bacterial classes with high abundance including *Gammaproteobacteria*, *Actinobacteria*, *Bacilli*, *Bacteroidia*, *Clostridia*, *Betaproteobacteria*, *Tissierellia*, *Flavobacteriia*, *Alphaproteobacteria*, *Thermoleophilia*, *Cytophagia*, *Sphingobacteriia*, *Negativicutes*, and *Verrucomicrobiae*. Composition of the classes indicated that *Gammaproteobacteria* was the dominant bacterial class in all locations, except one, where *Bacilli* had a high relative abundance followed by *Gammaproteobacteria*, *Betaproteobacteria* and *Actinobacteria* (Supplementary Table 6).

Overall, *Francisellaceae*, *Corynebacteriaceae*, *Staphylococcaceae*, *Moraxellaceae*, *Flavobacteriaceae*, *Clostridiaceae*, *Muribaculaceae*, *Bacillaceae*, *Neisseriaceae*, *Enterobacteriaceae*, and *Pseudomonadaceae* were the predominant families (Fig. 1B; Supplementary Table 7). *Enterobacteriaceae* had a highest relative abundance of 79.22% while *Bacillaceae* had a high relative abundance of 54.74%. However, *Francisellaceae* was dominant in all locations, with highest relative abundance of up to 99.1% in one location. *Muribaculaceae* was found from most of the locations, however with low relative abundance.

The dominant bacterial genus was *Francisella*. It was recorded in all locations, comprising up to 99.1% of relative abundance in one location (Fig. 2B; Supplementary Table 8). The *Escherichia* showed a relative abundance of 48.41% followed by *Bacillus*, which had a relative abundance of 45.84% while *Siccibacter* had highest relative abundance of 30.81%. *Corynebacterium* was recorded in all locations; however, the *Clostridium* and *Flavobacterium* were recorded in samples from only two locations. Though, *Staphylococcus* was recorded in all locations it was in a low relative abundance (Fig. 2B).

### Microbial diversity in 2010 and 2019

Richness of tick microbiota (richness of taxa among the samples) associated with *H. dromedarii* in 2010 samples (371 OTUs) was higher compared to 2019 samples (191 OTUs).

**Figure 3:**
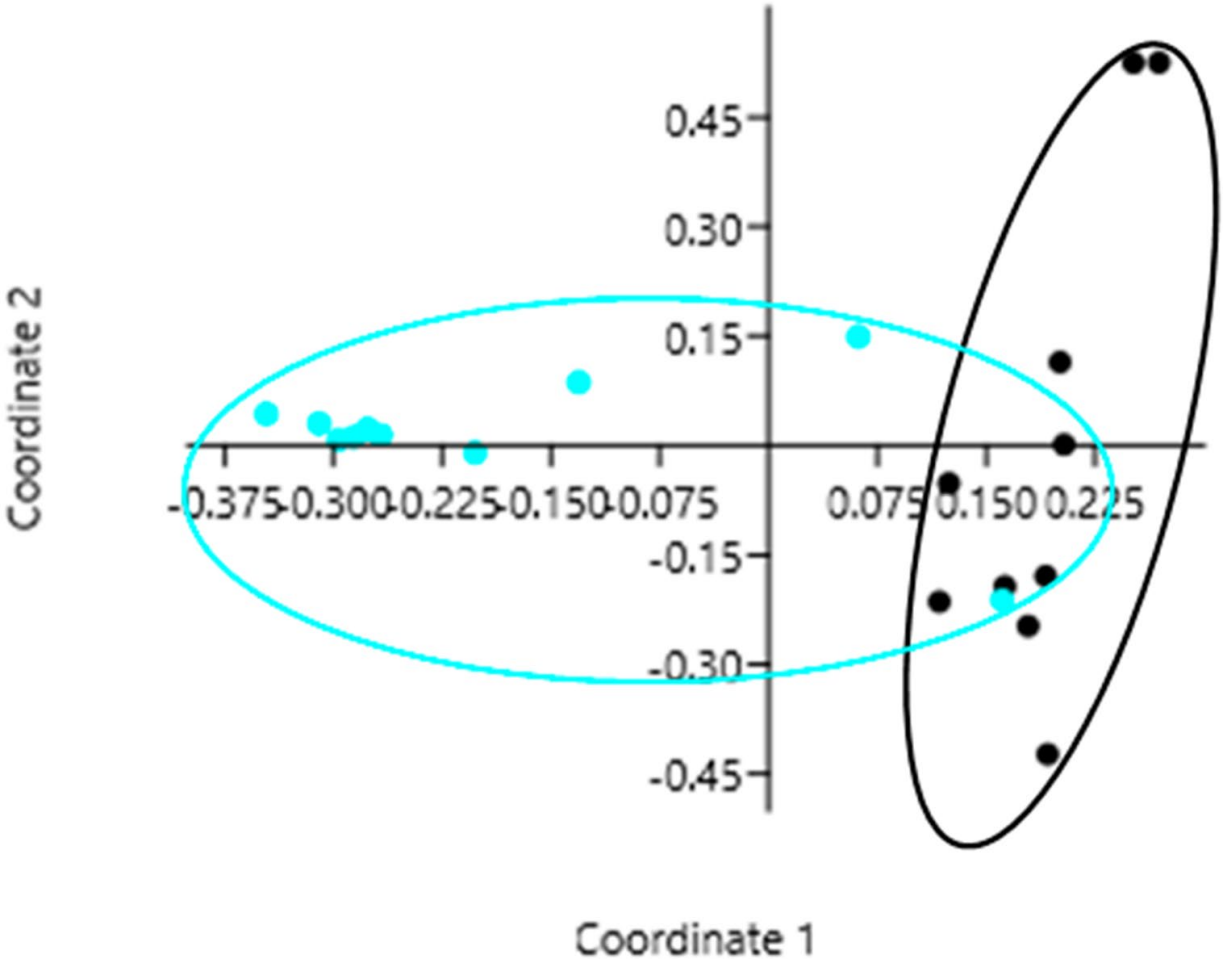
Principle Coordinates Analysis (PCoA) showing microbial diversity between years 2010 (black circles) and 2019 (blue circles).

Principle Coordinates Analysis showed that Coordinates 1, 2 and 3 accounted for over 84% of the variation (based on cumulative Eigenvalues) and the first two coordinates accounted for over 78% of the variation. Furthermore, there was a clear separation among the microbial communities between years with the exception of one site (Malaket, MQ) (Fig. 3). Samples from this site in 2019 had a microbial community more closely aligned with the microbial communities from 2010. However, the microbial community of the same site in 2010 was completely different (Fig. 3). Richness of genera differed significantly between years with higher richness recorded in 2010 (22.9 in 2010 versus 8.3 in 2019, two sample paired *t*-test, *p* < 0.0001). The Shannon Wiener index did not differ significantly between years (1.71 in 2010 versus 1.53 in 2019, two sample paired *t*-test, *p* > 0.05). In contrast, the Index of Evenness was significantly higher in 2010 (0.26 in 2010 versus 0.59 in 2019; two sample paired *t*-test, *t* = −6.27, *p* = 0.0001). The Index of Dominance (D) was not significantly different between years (0.27 in 2010 versus 0.27 in 2019; two sample paired *t*-test, *p* > 0.05).

### Associations between bacterial genera

**Figure 4:**
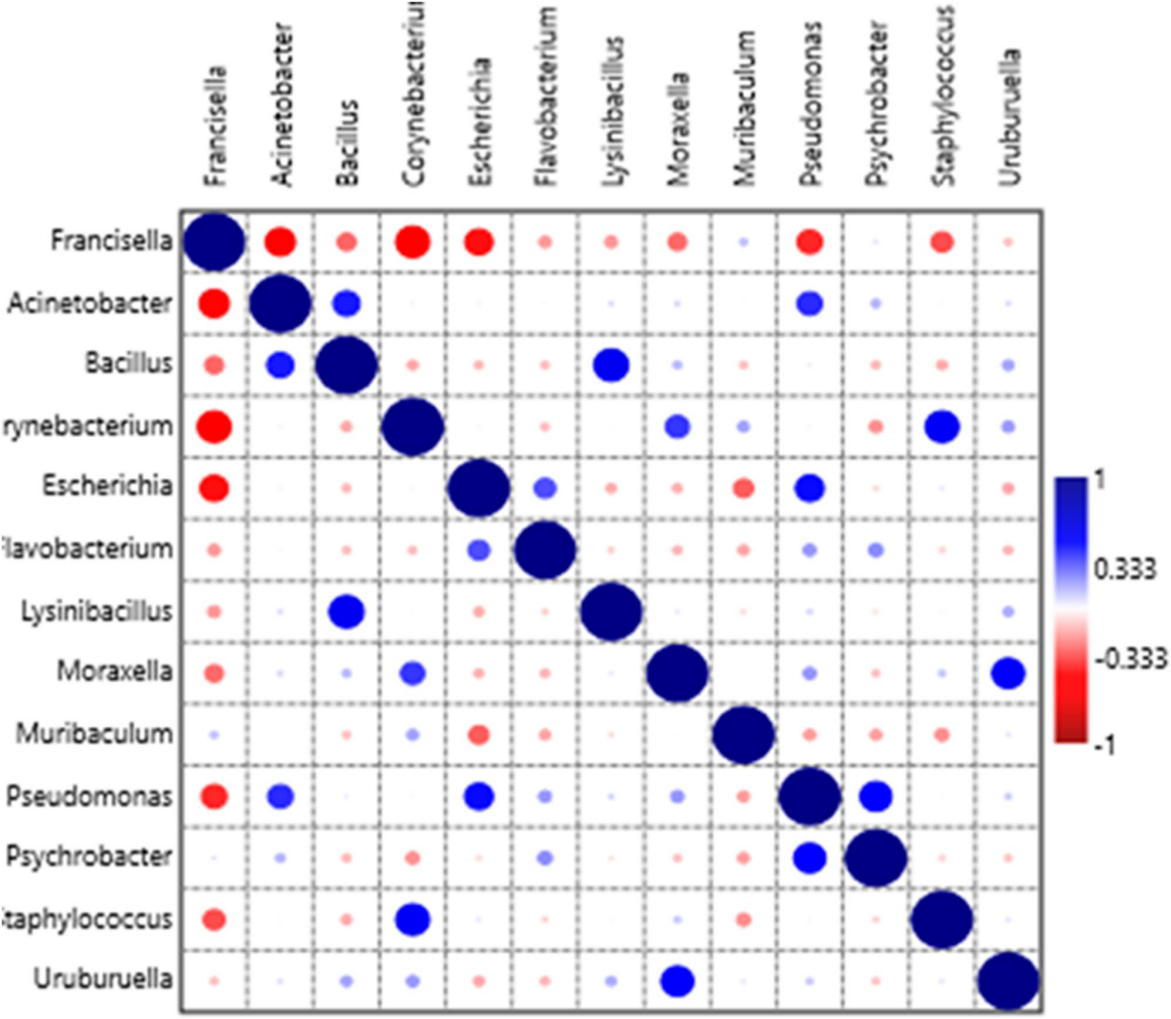
Pearson’s correlation coefficients indicating associations between bacterial genera showing significantly positive interactions (large dark blue circles) and significantly negative interactions (large red circles).

Pearson’s correlation coefficients (*r*) indicated that many bacterial genera were significantly correlated with each other (Fig. 4; Supplementary Table 9). *Francisella* was significantly negatively correlated with *Acinetobacter*, *Corynebacterium* and *Escherichia*. *Bacillus* was significantly positively correlated with *Lysinibacillus*. *Staphyllococcus* and was positively correlated with *Corynebacterium*. *Escherichia* was significantly positively correlated with *Pseudomonas* and *Moraxella* was correlated with *Uruburuella*. *Acinetobacter*, *Fransicella* and *Escherichia* were significant predictors of many bacterial genera (Table 1). *Acinetobacter* counts were significantly predicted by *Corynebacterium*, *Escherichia*, *Francisella*, *Lysinibacillus*, *Moraxella*, *Pseudomonas* and *Psychrobacter*. Similarly, *Escherichia* counts were significantly predicted by *Acinetobacter*, *Corynebacterium*, *Francisella*, *Lysinibacillus*, *Moraxella*, *Pseudomonas* and *Psychrobacter.* On the hand, *Francisella* counts were significantly predicted by *Acinetobacter* and *Escherichia*.

## Discussion

Camel ticks can carry and transmit potential pathogens^6,49–53^. Microbial diversity in ticks plays a significant role in pathogen transmission, vector competence^54,55^, and tick reproductive fitness^56^. Tick-borne pathogens can significantly decrease the production of camel milk and meat and may affect the racing breeds. We found a diverse array of pathogens in *H. dromedarii* ticks, highlighting the reservoir potential of this tick species for significant pathogens.

The patterns of bacterial phyla in the current study were consistent with findings of Elbir et al*.*^48^ where the *Proteobacteria* was the most abundant followed by *Firmicutes* and *Actinobacteria*. In addition, they were consistent with the results of Thapa et al*.*^43^, who also found *Proteobacteria* with the highest relative abundance across all baseline *Ixodes scapularis* male and female ticks under different temperatures in the USA. In similar studies, the bacterial phylum *Proteobacteria *was reported to be the most dominant (89%) in the microbiota of whole *Amblyomma tuberculatum* ticks infesting the gopher tortoise^57^ and (83.39%) in bacterial communities associated with *Amblyomma maculatum*^58^. In addition, it was found to be the overall dominant phylum followed by *Actinobacteria* and *Firmicutes* in *Ixodes ricinus* ticks on sheep in Northern Italy^37^. Overall, these findings indicate that *Proteobacteria* is a very common phylum, which exists in different tick species.

Bacterial classes (16 classes in 2010 and 14 in 2019) observed in this study were comparable to Khoo et al.^40^, where the taxonomical composition of tick samples indicated that the abundant bacterial class was *Gammaproteobacteria* along with *Alphaproteobacteria*, *Actinobacteria*, *Bacilli* and *Deltaproteobacteria*, which represented 80% to 99% of the population in each of the samples. However, Karim et al*.*^41^ documented predominantly only six classes namely *Bacilli*, *Gammaproteobacteria*, *Betaproteobacteria*, *Clostridia*, *Alphaproteobacteria* and *Actinobacteria*, after profiling of the bacteria sampled from tick species collected from various livestock. In another study, the bacterial DNA sequences of *Alphaproteobacteria* and *Gammaproteobacteria* types were abundant in *Ixodes persulcatus*, *Ixodes pavlovskyi*, and *Dermacentor reticulatus* samples with 30.2% and 60.8% average occurrence, respectively^59^. Generally, these studies in addition to the current study show that the above-mentioned common bacterial classes have a wide geographical distribution occurring in ticks from the UAE, Malaysia, Pakistan and Russia.

Patterns of abundance of bacterial families in this study differed from the findings of Karim et al*.*^41^ who found *Oxalobacteraceae*, *Staphylococcaceae*, *Clostridiaceae*, *Enterobacteriaceae*, *Coxiellaceae*, *Rickettsiaceae*, *Streptococcaceae*, and *Lactobacillaceae* as the predominant microbial families in tick samples that were not *H. dromedarii*. Some of this variation can be explained in light of quantitative and qualitative differences in microbial communities between hosts^41,67^. *Enterobacteriaceae* was the most abundant bacterial family in *R. microplus* ticks collected from cattle whereas *Rickettsiaceae*, *Oxalobacteraceae*, and *Micrococcaceae* were abundant in the *R*. *turanicus* ticks infesting goats^41^. Furthermore, the results of this study differ from Ravi et al*.*^60^ who reported four bacterial families: *Coxiellaceae*, *Francisellaceae*, *Rickettsiaceae* and *Anaplasmataceae* in *H. dromedarii*, *Rhipicephalus sanguineus* and *Haemaphysalis concinna*. The differences among families could be attributed to host specific factors. The existence of common families among different tick hosts may indicate that these bacterial families are generalists and may not require a very specific host internal environment. Our results are partly consistent with the findings of Kurilshikov et al*.*^59^ who found *Francisellaceae* as the most abundant family in *Dermacentor reticulatus* ticks and the *Moraxellaceae* in *Ixodes persulcatus*. In addition, Budachetri et al*.*^58^ reported that *Francisellaceae* and *Enterobacteriaceae* were the prevalent bacterial families in *Amblyomma maculatum* ticks. Based on these findings, *Francisellaceae* and *Enterobacteriaceae* coexist in *H. dromedarii* and *A. maculatum* suggesting that they thrive under similar conditions and microbial interactions inside the host. In general, the composition of microbial families can be affected by external and stochastic factors, which contribute to producing high or low diversity inside each individual tick. Although in this study, we pointed out the diversity in microbial families within *H. dromedarii*, however we did not identify the environmental and host-related factors, which might shape this complex microbial ecosystem. We assume that certain interactions among microorganisms inside *H. dromedarii* result in the dominance of some families over the others.

The use of 16S rRNA gene for identification of a broad range of clinically relevant bacterial pathogens is a good tool to assess microbial communities. However, short-read sequencing platforms which target different regions of 16S rRNA do not provide good taxonomic resolution when compared to sequencing the entire gene^61^. This implies that 16S rRNA gene-based identification is reliable up to the genus level. In addition, getting full-length or near full-length 16S sequences are crucial for making confident genus level taxonomic placements. Therefore, the genus level identifications presented in the current study are provided as preliminary baseline data, which may require further confirmation. Our results indicated that *Acinetobacter* and *Corynebacterium* were the two most abundant genera detected in the microbiota of *H. dromedarii* from all locations with high sequence ratios among a total of 31 genera in 2010 samples with more than 1% or some with 1% sequence reads at different locations. Other abundant genera included *Escherichia*, *Proteus*, *Staphylococcus*, *Bacillus*, *Flavobacterium* and *Stenotrophomonas*. However, the *Francisella* was the dominant genus (99.1%) in 2019 among all 15 abundant genera. *Bacillus*, *Escherichia*, *Siccibacter* and *Acinetobacter* were the other predominant genera at different locations in 2019 samples. The genus *Francisella* was confirmed previously in *H. dromedarii* ticks from Palestine^47^ and Saudi Arabia^48^.

We found significant associations between *Acinetobacter*, *Escherichia* and *Francisella* and between these three genera and several other genera. Little is known about *Francisella* and their associations with ticks. It appears that many diverse bacterial genera co-exist with tick-borne pathogens^62^ and endosymbiotic forms could increase colonization potential of pathogenic forms^63^. *Francisella* appears to occur in many ticks species, most commonly in mutualistic forms^62^. However, phylogenetic similarities between mutualistic and pathogenic *Francisella* suggest periodic and perhaps even frequent shifts from non-pathogenic forms^62,64^. Nonetheless, the co-occurrence of non-pathogenic and pathogenic bacteria may not always result in genetic transformations^65^, suggesting that multiple factors could influence pathogenicity in tick microbiota. Moreover, the constant occurrence of the *Francisella* indicates a systemic association between arthropods and this bacterial genus. Our finding of negative associations between *Francisella*, *Acinetobacter* and *Escherichia* could indicate possible suppressive effects of the former on the latter two genera. On the other hand, positive association between *Acinetobacter* and a broad range of bacterial genera also deserves further consideration. Many species of *Acinetobacter* are known to be pathogenic, while others are considered commensal and even part of the normal flora of animals^66^.

It is important to recognize that *Francisella* was reported in 2010 and 2019 and was found with the highest abundance (99%) in 2019. This finding is consistent with the overall change in bacterial communities experienced in 2019, with the general rise in *Francisella*. If future studies confirm the presence of pathogenic genus, *Francisella* in the UAE, this could be a potential emerging disease pathogen in the country and may affect the people who are closely working with the camels such as workers at farms and slaughter houses, veterinary hospitals and research centers. Again, we emphasize that the above-mentioned bacterial genera and species need further confirmation.

In conclusion, the present study advances our knowledge about the microbial communities in *H. dromedarii* ticks. It provides clear evidence that the microbiota of *H. dromedarii* is rich and diverse with a potential of harboring pathogenic bacteria, which pose a serious health risk to camels and people. Overall, the 16S rRNA gene-based sequencing, presented in the current study, gives excellent phylum, class, and family identifications and sheds light on the microbial diversity in *H. dromedarii* in general. Additionally, it gives baseline genus identification considering some of the limitations of 16S rRNA gene-based sequencing and consequently these findings should serve as foundation for future studies. Existing evidence warrants further investigation of the microbial ecology of the *H. dromedarii* and calls for deeper understanding of how some species of its microbiota become dominant over time especially the pathogenic ones. Our results set the stage for further screening and detection (through active surveillance) of pathogenic genera and species that pose serious health risks to camels and people. Moreover, more research is required to investigate the functional and the ecological implications of the bacterial communities associated with *H. dromedarii*.

## Methods

### Tick collection

In a cross-sectional study, we collected ticks manually from camels in 2010 and 2019. In 2010, we completed a project in which we collected large number of *H. dromedarii* ticks and stored them in − 80 °C. In 2019, we started a new project on this tick species and we were keen to collect ticks from the same locations sampled in 2010 so that we could make a comparison of microbial communities between the samples collected in both projects and detect changes over time. Farms and camels were selected randomly. In 2010, we collected ticks from 10 locations (Al-Wagan, Al-Yahar, Bede’ Fares, Bede’Bent Suod, Dubai Road, Dwar Al-Shahenat, Malaket, Omghafa, Remah, and Swehan) in Al-Ain area at the eastern part of the UAE. In each location, we selected five camels, and from each camel we collected 10 ticks. In the laboratory, one partially engorged female tick was picked out of the 10 ticks collected per animal to be subjected to DNA extraction and sequencing. The same strategy of tick sampling was followed in 2019. As a result, we gathered 1000 ticks in total in 2010 and 2019 and from them we used 100 partially engorged female ticks at the rate of 50 ticks each year. Ticks were kept in plastic vials (50 ml) in − 80 °C freezer until DNA extraction. Tick collection was carried out in strict accordance with the recommendations of the Animal Research Ethics Committee (A-REC) of the UAE University (ethical approval# ERA_2019_5953). In addition, the experimental protocol was approved by the UAE University Research Office.

### Tick identification, genomic DNA extraction and pooling

The identification of ticks as *H*. *dromedarii* was done morphologically using the keys of Apanaskevich et al.^67^ and Walker et al.^68^ and based on DNA sequencing using cytochrome oxidase subunit I (COI) gene^49^. Briefly, in the males the sub-anal plates are aligned outside the adanal plates. In addition, the adanal plates have a characteristic shape with both long margins strongly curved in parallel. In the females, the genital aperture has posterior lips with a narrow V, which is also found in *Hyalomma impeltatum*, but the posterior margin of their scutum is distinctly sinuous compared to a slightly sinuous margin in *H. dromedarii*. With molecular identification, a segment of the COI gene was amplified in polymerase chain reaction using a primer pair Fish1F: 5′-TCAACCAACCACAAAGACATTGGCAC-3′ and Fish1R: 5′-TAGACTTCTGGGTGGCCAAAGAATCA-3′^69^ under the following thermocycling conditions: 2 min at 95 °C followed by 30 cycles of 1 min at 94 °C, 1 min at 54 °C, and extension for 90 s at 72 °C. We already mentioned that in each sampling year, we collected 50 partially engorged female ticks from which we extracted DNA individually. Before DNA extraction, each tick was thoroughly washed with distilled water. Each whole tick was crushed manually using a sterile Kimble Kontes pellet pestle (Thermo Fisher, Waltham, MA) inside a sterile 1.5 ml microcentrifuge tube. Genomic DNA was extracted from each individual tick using QIAamp Tissue Kit (Qiagen, Hilden, Germany) following the manufacturer’s protocol. Quality and concentration of the extracted DNA was determined with a spectrophotometer (Nano Drop ND-1000, Erlangen, Germany). In addition, DNA quality was assessed on a 1% agarose gel stained with ethidium bromide and visualized under UV light. DNA was stored in a − 20 °C freezer until used. Prior to sequencing, extracted DNA samples from individual ticks were pooled according to collection location. This resulted in having 10 DNA pools for each sampling year.

### 16S rRNA gene amplicon sequencing and bioinformatics analysis

To evaluate the microbial communities in camel ticks, a 16S rRNA gene-based analysis was conducted. A total of 20 DNA pooled samples were shipped to Macrogen Inc (Seoul, South Korea). The following primers were used for amplifying the V3 V4 region: Bakt_341F: CCTACGGGNGGCWGCAG Bakt_805R: GACTACHVGGGTATCTAATCC^70^ using the Herculase II Fusion DNA polymerase Nextera XT Index Kit V2. Sequencing was performed on a Illumina MiSeq platform with read length of 301 bp. Demultiplexed paired-end sequence reads in FASTQ format for each sample were merged using fast length adjustment of short reads (FLASH) version 1.2.11^71^. Next, CD-HIT-OTU^72^ was used to cluster the reads from 2010 and 2019 into OTUs using default options. CD-HIT-OTU filters out low quality reads, trims extra-long tails, identifies chimeric reads and clusters reads into OTUs with a cutoff of 97% identity. Finally, taxonomic assignment of OTUs was performed using the *assign_taxony.py* script from QIIME 1.9.1^73^ by performing a Basic Local Alignment Search Tool (BLAST)^74^ search against the National Center for Biotechnology Information (NCBI) 16S microbial database. Taxonomic levels of bacteria from phylum to genus were profiled in samples across all locations. Taxonomic abundance ratios were calculated from taxonomic abundance count to summarize and interpret the results at phylum, class, family and genus level. Sequences were deposited in NCBI Sequence Read Archive under the BioProject ID PRJNA639925.

### Quantification and statistical analyses

We conducted Principle Coordinates Analysis (PCoA) to determine patterns of diversity in bacterial communities. The PCoA were conducted and visualized using the software PAST 5.27 Paleontological statistics software package^75^ (Øyvind Hammer, Natural History Museum, University of Oslo, Norway, ohammer@nhm.uio.no ). OTU count of each genus was entered and the samples were categorized by year (2010 and 2019). Eigenvalues were examined to determine the extent of variation explained by the first three principle coordinates (Coordinates 1–3)^76^. We calculated different indices of diversity since a single index often does not reflect the true nature of diversity and a combination provides an approximation of diversity. We estimated Richness (total number of genera, based on OTUs obtained for each genus); Shannon Wiener Index; and the Index of Dominance. The Shannon Wiener Index of diversity was calculated using the following formula:

$$ {\text{Shannon}{-}\text{Wiener}}\,{\text{Index}}\,H = - \mathop \sum \limits_{i}^{S} p_{i} \log p_{i} $$

where S—the total number of genera, *i*—the number of OTUs for genus i; and *pi*—relative proportion of genus *i*.

Index of Evenness (relative abundance of each genus, based on OTUs) was calculated as follows:

$$ {\text{Index}}\,{\text{of}}\,{\text{Evenness}},\,{\text{E}} = {\text{e}}^{H} /{\text{S}} $$

where *H*—Shannon–Weiner’s Index and S is the total number genera.

The Index of Dominance (D) was calculated using the following formula:

$$ {\text{D}} = {\text{number}}\,{\text{of}}\,{\text{OTUs}}\,{\text{for}}\,{\text{the}}\,{\text{dominant}}\,{\text{genera/the}}\,{\text{total}}\,{\text{number}}\,{\text{of}}\,{\text{OTUs}}. $$

We compared all these indices between years using paired two sample *t*-test using PAST^75^. Pearson’s Correlation Coefficient (*r*) was calculated to determine associations between different genera that occurred in 2010 and 2019^77^. Genera with significant correlations were subjected to stepwise regression analysis, with backward selection^77^. One genus was used as the response variable and all other genera that had significant correlations with response variable were used as explanatory variables. Genera were removed individually based on significance to see the effect on the overall model. Only those genera that improved the overall model were retained, while genera did not affect the model were removed. The process was repeated with each genus that had a significant correlation with other genera. For all tests, the value of α was set at 0.05.

## Supplementary information

Supplementary information.
