# Supplementary material for: Microbial communities associated with the camel tick, Hyalomma dromedarii: 16S rRNA gene-based analysis
Source: Sci Rep. 2020 Oct 12;10:17035. doi: 10.1038/s41598-020-74116-7 (PMC7550333; doi:10.1038/s41598-020-74116-7)
Supplement: Supplementary file 1 — Supplementary information. [file 41598_2020_74116_MOESM1_ESM.docx]

**Microbial communities associated with the camel tick, *Hyalomma dromedarii*: 16S rRNA gene-based analysis**

Nighat Perveen, Sabir Bin Muzaffar, Ranjit Vijayan, Mohammad Ali Al-Deeb*

**S1 Table.** Microbial phyla (presence in %) detected in *H. dromedarii* adult ticks from ten locations in Al-Ain, UAE in 2010.

| Phylum | BF | BS | DR | DS | MQ | RH | SW | OM | AW | AY |
| --- | --- | --- | --- | --- | --- | --- | --- | --- | --- | --- |
| Actinobacteria | 7.40 | 1.51 | 18.25 | 41.60 | 2.25 | 17.66 | 29.06 | 26.12 | 21.68 | 33.84 |
| Bacteroidetes | 26.95 | 0.87 | 1.11 | 1.30 | 0.37 | 0.71 | 0.60 | 0.26 | 3.82 | 0.46 |
| Chloroflexi | 0.00 | 0.00 | 0.00 | 0.02 | 0.00 | 0.00 | 0.00 | 0.00 | 0.00 | 0.00 |
| Cyanobacteria | 0.00 | 0.00 | 0.07 | 0.00 | 0.00 | 0.00 | 0.00 | 0.00 | 1.15 | 0.07 |
| Deinococcus-Thermus | 0.00 | 0.00 | 0.00 | 0.00 | 0.00 | 0.00 | 0.00 | 0.00 | 0.00 | 0.02 |
| Firmicutes | 6.15 | 43.38 | 30.19 | 17.98 | 9.52 | 8.05 | 6.92 | 30.74 | 47.27 | 19.26 |
| Gemmatimonadetes | 0.00 | 0.00 | 0.00 | 0.00 | 0.00 | 0.01 | 0.00 | 0.03 | 0.00 | 0.00 |
| Planctomycetes | 0.00 | 0.00 | 0.00 | 0.00 | 0.00 | 0.00 | 0.00 | 0.28 | 0.00 | 0.00 |
| Proteobacteria | 59.47 | 54.21 | 50.15 | 37.97 | 87.86 | 73.57 | 63.28 | 42.53 | 25.21 | 46.30 |
| Verrucomicrobia | 0.00 | 0.00 | 0.00 | 0.41 | 0.00 | 0.00 | 0.13 | 0.00 | 0.21 | 0.00 |

**S2 Table.** Microbial classes (presence in %) detected in *H. dromedarii* adult ticks from ten locations in Al-Ain, UAE in 2010

| Class | BF | BS | DR | DS | MQ | RH | SW | OM | AW | AY |
| --- | --- | --- | --- | --- | --- | --- | --- | --- | --- | --- |
| Actinobacteria | 7.40 | 1.51 | 18.24 | 41.60 | 2.25 | 17.66 | 29.06 | 26.12 | 21.62 | 33.84 |
| Alphaproteobacteria | 0.07 | 0.00 | 0.10 | 0.36 | 0.08 | 0.14 | 0.05 | 0.26 | 0.48 | 0.07 |
| Bacilli | 4.52 | 43.36 | 26.24 | 11.77 | 8.89 | 6.43 | 2.58 | 30.07 | 41.90 | 16.69 |
| Bacteroidia | 0.07 | 0.07 | 0.39 | 0.99 | 0.13 | 0.48 | 0.38 | 0.06 | 3.43 | 0.22 |
| Betaproteobacteria | 0.15 | 0.04 | 8.87 | 0.58 | 0.52 | 0.42 | 0.13 | 6.63 | 0.12 | 3.35 |
| Chitinophagia | 0.01 | 0.00 | 0.00 | 0.30 | 0.00 | 0.00 | 0.00 | 0.08 | 0.00 | 0.00 |
| Clostridia | 0.53 | 0.02 | 0.21 | 0.62 | 0.12 | 0.05 | 0.05 | 0.13 | 3.95 | 0.31 |
| Coriobacteriia | 0.00 | 0.00 | 0.01 | 0.00 | 0.00 | 0.00 | 0.00 | 0.00 | 0.06 | 0.00 |
| Cytophagia | 0.00 | 0.00 | 0.00 | 0.00 | 0.00 | 0.00 | 0.00 | 0.02 | 0.00 | 0.00 |
| Deinococci | 0.00 | 0.00 | 0.00 | 0.00 | 0.00 | 0.00 | 0.00 | 0.00 | 0.00 | 0.02 |
| Deltaproteobacteria | 0.00 | 0.00 | 0.00 | 0.00 | 0.00 | 0.00 | 0.00 | 0.00 | 0.15 | 0.00 |
| Erysipelotrichia | 0.00 | 0.00 | 0.00 | 0.12 | 0.04 | 0.00 | 0.00 | 0.05 | 0.02 | 0.00 |
| Flavobacteriia | 26.66 | 0.80 | 0.72 | 0.00 | 0.01 | 0.23 | 0.09 | 0.11 | 0.39 | 0.23 |
| Fusobacteriia | 0.00 | 0.00 | 0.00 | 0.00 | 0.00 | 0.00 | 0.00 | 0.00 | 0.00 | 0.00 |
| Gammaproteobacteria | 59.25 | 54.17 | 41.18 | 37.03 | 87.25 | 73.01 | 63.10 | 35.65 | 24.45 | 42.89 |
| Gemmatimonadetes | 0.00 | 0.00 | 0.00 | 0.00 | 0.00 | 0.01 | 0.00 | 0.00 | 0.00 | 0.00 |
| Longimicrobia | 0.00 | 0.00 | 0.00 | 0.00 | 0.00 | 0.00 | 0.00 | 0.03 | 0.00 | 0.00 |
| Negativicutes | 0.05 | 0.00 | 0.08 | 0.00 | 0.00 | 0.09 | 0.00 | 0.00 | 0.17 | 0.00 |
| Oligoflexia | 0.00 | 0.00 | 0.00 | 0.00 | 0.00 | 0.00 | 0.00 | 0.00 | 0.01 | 0.00 |
| Planctomycetia | 0.00 | 0.00 | 0.00 | 0.00 | 0.00 | 0.00 | 0.00 | 0.28 | 0.00 | 0.00 |
| Sphingobacteriia | 0.21 | 0.00 | 0.00 | 0.00 | 0.22 | 0.00 | 0.13 | 0.00 | 0.00 | 0.00 |
| Thermomicrobia | 0.00 | 0.00 | 0.00 | 0.02 | 0.00 | 0.00 | 0.00 | 0.00 | 0.00 | 0.00 |
| Tissierellia | 1.05 | 0.00 | 3.65 | 5.47 | 0.47 | 1.48 | 4.29 | 0.50 | 1.23 | 2.27 |
| Verrucomicrobiae | 0.00 | 0.00 | 0.00 | 0.41 | 0.00 | 0.00 | 0.13 | 0.00 | 0.21 | 0.00 |

**S3 Table.** Microbial families (presence in %) detected in *H. dromedarii* adult ticks from ten locations in Al-Ain, UAE in 2010

| Family | AW | AY | BF | BS | DR | DS | MQ | OM | RH | SW |
| --- | --- | --- | --- | --- | --- | --- | --- | --- | --- | --- |
| Moraxellaceae | 2.84 | 13.43 | 14.27 | 49.72 | 31.02 | 33 | 77.52 | 16.62 | 7.1 | 2.31 |
| Corynebacteriaceae | 21 | 32.23 | 7.25 | 1.42 | 14.2 | 36.62 | 2.04 | 25 | 13.28 | 23.82 |
| Enterobacteriaceae | 9.51 | 1.8 | 23.54 | 3.47 | 7 | 0.5 | 7.46 | 4.3 | 54.63 | 4.54 |
| Flavobacteriaceae | 0.22 | 0.23 | 26.66 | 0.8 | 0.72 | 0 | 0.01 | 0.04 | 0.23 | 0.1 |
| Staphylococcaceae | 38.1 | 15 | 3.74 | 0.28 | 1.5 | 6.42 | 5.84 | 19.3 | 6.01 | 1.81 |
| Bacillaceae | 1 | 1.03 | 0.37 | 37.33 | 24.5 | 3 | 2.54 | 6.06 | 0.1 | 0.3 |
| Morganellaceae | 0 | 0.3 | 19.73 | 0 | 1 | 3 | 0 | 0.02 | 6.03 | 55.82 |
| Francisellaceae | 11.4 | 2.33 | 0.86 | 1 | 0.34 | 0.2 | 0.33 | 13 | 2.88 | 0.33 |
| Bacteroidaceae | 2.3 | 0.12 | 0 | 0 | 0 | 0 | 0 | 0 | 0.28 | 0.04 |
| Ruminococcaceae | 1.4 | 0 | 0 | 0 | 0 | 0 | 0 | 0 | 0 | 0 |
| Peptoniphilaceae | 1.13 | 2.24 | 1.05 | 0 | 3.6 | 5.4 | 0.47 | 0.5 | 1.48 | 4.3 |
| Lachnospiraceae | 1.12 | 0 | 0.44 | 0.02 | 0 | 0.31 | 0.05 | 0.05 | 0.03 | 0.01 |
| Planococcaceae | 1.04 | 0 | 0 | 0 | 0 | 0.01 | 0.14 | 3 | 0 | 0.03 |
| Aerococcaceae | 1 | 0.23 | 0.1 | 0 | 0.1 | 2.41 | 0.25 | 0.3 | 0.28 | 0.4 |
| Actinomycetaceae | 0.4 | 0.3 | 0.1 | 0 | 1.1 | 0 | 0 | 0.22 | 0.52 | 0.8 |
| Pseudomonadaceae | 0.4 | 0.54 | 0.8 | 0.02 | 1.6 | 0.5 | 1.7 | 0.1 | 2.32 | 0.1 |
| Brevibacteriaceae | 0.2 | 1.1 | 0.1 | 0 | 2.3 | 3.6 | 0.05 | 0.1 | 3.42 | 2.76 |
| Enterococcaceae | 0.19 | 0.4 | 0.28 | 5.67 | 0.12 | 0.17 | 0.11 | 0.8 | 0.05 | 0.04 |
| Pasteurellaceae | 0.03 | 0 | 0.01 | 0 | 0.21 | 0.16 | 0.1 | 2 | 0 | 0 |
| Neisseriaceae | 0.01 | 0.34 | 0.13 | 0 | 8 | 0.42 | 0.32 | 7 | 0.18 | 0 |
| Xanthomonadaceae | 0 | 24.5 | 0.07 | 0 | 0.03 | 0.02 | 0 | 0.02 | 0 | 0 |
| Comamonadaceae | 0 | 3 | 0 | 0.04 | 0 | 0 | 0.01 | 0 | 0.09 | 0 |
| Dermabacteraceae | 0 | 0.19 | 0.02 | 0 | 0.21 | 0.43 | 0.01 | 0.5 | 0.32 | 1.25 |
| Muribaculaceae | 0 | 0 | 0.07 | 0 | 0.23 | 1 | 0.13 | 0 | 0 | 0.15 |
| Alcaligenaceae | 0 | 0 | 0.02 | 0 | 1 | 0.16 | 0.19 | 0 | 0.15 | 0.13 |
| Others | 6.71 | 0.69 | 0.39 | 0.23 | 1.22 | 2.67 | 0.73 | 1.07 | 0.62 | 0.96 |

**S4 Table.** Microbial genera (presence in %) detected in *H. dromedarii* adult ticks from ten locations in Al-Ain, UAE in 2010

| Genus | Aw | AY | BF | BS | DR | DS | MQ | OM | RH | SW |
| --- | --- | --- | --- | --- | --- | --- | --- | --- | --- | --- |
| Acinetobacter | 2.5 | 5.92 | 14.2 | 49.72 | 24.55 | 32.08 | 75.66 | 15.81 | 7.09 | 2.31 |
| Escherichia | 7.52 | 1.57 | 23 | 3.43 | 3.36 | 0 | 6.41 | 0.11 | 53.13 | 4.37 |
| Corynebacterium | 20.73 | 32.23 | 7.25 | 1.42 | 14.17 | 36.62 | 2.04 | 24.88 | 13.28 | 23.82 |
| Staphylococcus | 37.68 | 14.73 | 3.66 | 0.28 | 1.48 | 6.1 | 5.82 | 16.11 | 6 | 1.79 |
| Bacillus | 0.85 | 0.17 | 0.06 | 37.32 | 23.44 | 2.04 | 2 | 3.6 | 0.06 | 0.01 |
| Proteus | 0 | 0.26 | 19.73 | 0 | 0.78 | 2.73 | 0 | 0.02 | 6.03 | 55.82 |
| Flavobacterium | 0 | 0 | 26.65 | 0.8 | 0 | 0 | 0 | 0.04 | 0.23 | 0.09 |
| Francisella | 11.38 | 2.33 | 0.86 | 0.97 | 0.34 | 0.19 | 0.33 | 12.73 | 2.88 | 0.33 |
| Moraxella | 0.2 | 7.47 | 0.05 | 0 | 6.48 | 0.81 | 0.68 | 0.51 | 0.01 | 0.01 |
| Uruburuella | 0.01 | 0.34 | 0.13 | 0 | 7.94 | 0.42 | 0.32 | 6.43 | 0.18 | 0 |
| Stenotrophomonas | 0 | 24.5 | 0.04 | 0 | 0 | 0 | 0 | 0.02 | 0 | 0 |
| Brevibacterium | 0.19 | 1.05 | 0.07 | 0 | 2.25 | 3.55 | 0.05 | 0.1 | 3.42 | 2.76 |
| Enterococcus | 0.19 | 0.35 | 0.28 | 5.67 | 0.12 | 0.17 | 0.11 | 0.78 | 0.05 | 0.04 |
| Enterobacter | 1.98 | 0.2 | 0.47 | 0.03 | 3.62 | 0.45 | 0.68 | 4.12 | 1.47 | 0.17 |
| Helcococcus | 0.52 | 1.86 | 0.16 | 0 | 3.03 | 3.35 | 0.46 | 0.5 | 1.04 | 1.29 |
| Comamonas | 0 | 3 | 0 | 0 | 0 | 0 | 0.01 | 0 | 0.09 | 0 |
| Solibacillus | 1.04 | 0 | 0 | 0 | 0 | 0 | 0.14 | 2.61 | 0 | 0 |
| Jeotgalicoccus | 0.26 | 0.1 | 0.08 | 0 | 0 | 0.27 | 0 | 2.49 | 0 | 0.02 |
| Pseudomonas | 0.39 | 0.45 | 0.77 | 0.02 | 1.56 | 0.48 | 1.7 | 0.1 | 2.32 | 0.09 |
| Facklamia | 0.56 | 0 | 0.03 | 0 | 0 | 1.33 | 0.1 | 0.25 | 0 | 0 |
| Brachybacterium | 0 | 0.19 | 0.02 | 0 | 0.21 | 0.43 | 0.01 | 0.47 | 0.32 | 1.25 |
| Peptoniphilus | 0.16 | 0.2 | 0.23 | 0 | 0.42 | 0.16 | 0 | 0 | 0.18 | 1.18 |
| Lysinibacillus | 0.02 | 0.87 | 0.27 | 0.01 | 1.02 | 0.71 | 0.54 | 2.46 | 0.01 | 0.23 |
| Bacteroides | 2.3 | 0.12 | 0 | 0 | 0 | 0 | 0 | 0 | 0.28 | 0.04 |
| Mannheimia | 0 | 0 | 0.01 | 0 | 0.2 | 0.16 | 0.1 | 1.89 | 0 | 0 |
| Anaerococcus | 0.32 | 0.18 | 0.39 | 0 | 0.13 | 1.84 | 0 | 0 | 0.16 | 0.77 |
| Psychrobacter | 0.14 | 0.04 | 0 | 0 | 0 | 0.1 | 1.18 | 0.3 | 0 | 0 |
| Finegoldia | 0.03 | 0 | 0.26 | 0 | 0 | 0.03 | 0 | 0 | 0.08 | 1.05 |
| Trueperella | 0.36 | 0.25 | 0.03 | 0 | 1.05 | 0 | 0 | 0 | 0.52 | 0.67 |
| Ignavigranum | 0.07 | 0.22 | 0.08 | 0 | 0.08 | 1.04 | 0.14 | 0 | 0.22 | 0.39 |
| Muribaculum | 0 | 0 | 0.07 | 0 | 0.23 | 1 | 0.13 | 0 | 0 | 0.15 |
| Others | 10.6 | 1.4 | 1.15 | 0.33 | 3.54 | 3.94 | 1.39 | 3.67 | 0.95 | 1.35 |

**S5 Table.** Microbial phyla (presence in %) detected in *H. dromedarii* adult ticks from ten locations in Al-Ain, UAE in 2019

| Phylum | AW | AY | BF | BS | DR | DS | MQ | OM | RH | SW |
| --- | --- | --- | --- | --- | --- | --- | --- | --- | --- | --- |
| Actinobacteria | 0.26 | 5.04 | 0.19 | 0.45 | 7.90 | 0.28 | 0.31 | 0.53 | 0.82 | 0.07 |
| Bacteroidetes | 0.13 | 0.21 | 0.58 | 0.15 | 0.37 | 0.21 | 7.01 | 10.26 | 1.17 | 0.43 |
| Cyanobacteria | 0.02 | 0.00 | 0.00 | 0.00 | 0.08 | 0.00 | 0.00 | 0.00 | 0.30 | 0.00 |
| Firmicutes | 0.13 | 0.37 | 1.44 | 55.54 | 1.02 | 0.38 | 9.54 | 1.13 | 1.63 | 1.59 |
| Planctomycetes | 0.00 | 0.00 | 0.00 | 0.00 | 0.00 | 0.00 | 0.00 | 0.00 | 0.00 | 0.00 |
| Proteobacteria | 99.26 | 94.14 | 97.08 | 43.80 | 90.35 | 99.12 | 83.14 | 88.07 | 95.77 | 97.69 |
| Verrucomicrobia | 0.00 | 0.00 | 0.00 | 0.05 | 0.14 | 0.00 | 0.01 | 0.00 | 0.31 | 0.00 |

**S6 Table.** Microbial classes (presence in %) detected in *H. dromedarii* adult ticks from ten locations in Al-Ain, UAE in 2019

| Class | AW | AY | BF | BS | DR | DS | MQ | OM | RH | SW |
| --- | --- | --- | --- | --- | --- | --- | --- | --- | --- | --- |
| Actinobacteria | 0.26 | 5.04 | 0.17 | 0.45 | 7.90 | 0.28 | 0.31 | 0.53 | 0.82 | 0.07 |
| Alphaproteobacteria | 0.01 | 0.00 | 0.08 | 0.11 | 0.00 | 0.00 | 0.01 | 0.01 | 0.00 | 0.08 |
| Bacilli | 0.07 | 0.21 | 1.44 | 55.52 | 0.61 | 0.30 | 0.70 | 0.85 | 1.23 | 1.52 |
| Bacteroidia | 0.12 | 0.20 | 0.58 | 0.13 | 0.37 | 0.21 | 0.05 | 0.09 | 1.17 | 0.31 |
| Betaproteobacteria | 0.01 | 0.02 | 5.71 | 0.48 | 0.04 | 0.06 | 0.01 | 0.26 | 0.00 | 0.00 |
| Chitinophagia | 0.00 | 0.00 | 0.00 | 0.00 | 0.00 | 0.00 | 0.00 | 0.00 | 0.00 | 0.11 |
| Clostridia | 0.05 | 0.10 | 0.00 | 0.02 | 0.27 | 0.08 | 8.70 | 0.10 | 0.40 | 0.07 |
| Cytophagia | 0.00 | 0.00 | 0.00 | 0.01 | 0.00 | 0.00 | 0.00 | 0.00 | 0.00 | 0.00 |
| Erysipelotrichia | 0.00 | 0.00 | 0.00 | 0.00 | 0.00 | 0.00 | 0.00 | 0.00 | 0.00 | 0.00 |
| Flavobacteriia | 0.02 | 0.01 | 0.00 | 0.00 | 0.00 | 0.00 | 6.89 | 9.83 | 0.00 | 0.00 |
| Gammaproteobacteria | 99.24 | 94.12 | 91.29 | 43.21 | 90.31 | 99.06 | 83.12 | 87.81 | 95.77 | 97.61 |
| Hydrogenophilalia | 0.00 | 0.00 | 0.00 | 0.00 | 0.00 | 0.00 | 0.00 | 0.00 | 0.00 | 0.00 |
| Negativicutes | 0.00 | 0.00 | 0.00 | 0.00 | 0.00 | 0.00 | 0.07 | 0.00 | 0.00 | 0.00 |
| Phycisphaerae | 0.00 | 0.00 | 0.00 | 0.00 | 0.00 | 0.00 | 0.00 | 0.00 | 0.00 | 0.00 |
| Sphingobacteriia | 0.00 | 0.00 | 0.00 | 0.01 | 0.00 | 0.00 | 0.07 | 0.34 | 0.00 | 0.00 |
| Thermoleophilia | 0.00 | 0.00 | 0.02 | 0.00 | 0.00 | 0.00 | 0.00 | 0.00 | 0.00 | 0.00 |
| Tissierellia | 0.00 | 0.06 | 0.00 | 0.00 | 0.13 | 0.00 | 0.07 | 0.18 | 0.00 | 0.00 |
| Verrucomicrobiae | 0.00 | 0.00 | 0.00 | 0.05 | 0.14 | 0.00 | 0.01 | 0.00 | 0.31 | 0.00 |

**S7 Table.** Microbial families (presence in %) detected in *H. dromedarii* adult ticks from ten locations in Al-Ain, UAE in 2019

| Family | Aw | AY | BF | BS | DR | DS | MQ | OM | RH | SW |
| --- | --- | --- | --- | --- | --- | --- | --- | --- | --- | --- |
| Francisellaceae | 99.1 | 94.1 | 89.5 | 27.4 | 89.3 | 98 | 0.2 | 62.7 | 95.5 | 97.6 |
| Corynebacteriaceae | 0.16 | 5.03 | 0.11 | 0.44 | 7.9 | 0.26 | 0.28 | 0.33 | 0.79 | 0.07 |
| Staphylococcaceae | 0.07 | 0.2 | 0.28 | 0.28 | 0.41 | 0.23 | 0.06 | 0.38 | 0.59 | 1.4 |
| Moraxellaceae | 0.04 | 0.06 | 1.61 | 13.51 | 0.04 | 0 | 3.07 | 21.61 | 0.28 | 0.04 |
| Flavobacteriaceae | 0.02 | 0.01 | 0 | 0 | 0 | 0 | 6.9 | 9.83 | 0 | 0 |
| Clostridiaceae | 0 | 0 | 0 | 0 | 0 | 0 | 8.61 | 0 | 0.12 | 0 |
| Muribaculaceae | 0 | 0.2 | 0.32 | 0.13 | 0.17 | 0.21 | 0 | 0.02 | 0.81 | 0.18 |
| Bacillaceae | 0 | 0.01 | 1.17 | 54.74 | 0.16 | 0.07 | 0 | 0.4 | 0 | 0.12 |
| Neisseriaceae | 0 | 0 | 5.71 | 0.44 | 0.03 | 0 | 0 | 0 | 0 | 0 |
| Enterobacteriaceae | 0 | 0 | 0 | 1.34 | 0.01 | 0.89 | 79.22 | 0.36 | 0 | 0 |
| Pseudomonadaceae | 0 | 0 | 0 | 0.77 | 0.26 | 0 | 0.62 | 2.04 | 0 | 0 |
| Others | 0.61 | 0.39 | 1.3 | 0.95 | 1.72 | 0.34 | 1.04 | 2.33 | 1.91 | 0.59 |

**S8 Table.** Microbial genera (presence in %) detected in *H. dromedarii* adult ticks from ten locations in Al-Ain, UAE in 2019

| Genus | Aw | AY | BF | BS | DR | DS | MQ | OM | RH | SW |
| --- | --- | --- | --- | --- | --- | --- | --- | --- | --- | --- |
| Francisella | 99.1 | 94.1 | 89.5 | 27.4 | 89.3 | 98 | 0.2 | 62.7 | 95.5 | 97.6 |
| Corynebacterium | 0.16 | 5 | 0.11 | 0.44 | 7.9 | 0.26 | 0.28 | 0.33 | 0.8 | 0.1 |
| Staphylococcus | 0.1 | 0.2 | 0.3 | 0.3 | 0.4 | 0.23 | 0.1 | 0.4 | 0.45 | 1.4 |
| Moraxella | 0.04 | 0.1 | 1.1 | 0.1 | 0 | 0 | 0 | 0 | 0 | 0 |
| Uruburuella | 0 | 0 | 5.7 | 0.44 | 0.03 | 0 | 0 | 0 | 0 | 0 |
| Bacillus | 0 | 0.01 | 1.11 | 45.84 | 0.16 | 0.05 | 0 | 0.06 | 0 | 0.12 |
| Muribaculum | 0 | 0.2 | 0.32 | 0.13 | 0.17 | 0.21 | 0 | 0.02 | 0.81 | 0.18 |
| Pseudomonas | 0 | 0 | 0 | 0.77 | 0.26 | 0 | 0.62 | 2.04 | 0 | 0 |
| Acinetobacter | 0 | 0.01 | 0 | 13.42 | 0.04 | 0 | 1.87 | 15.1 | 0.17 | 0.02 |
| Escherichia | 0 | 0 | 0 | 1.34 | 0.01 | 0 | 48.41 | 0.02 | 0 | 0 |
| Siccibacter | 0 | 0 | 0 | 0 | 0 | 0.89 | 30.81 | 0.33 | 0 | 0 |
| Lysinibacillus | 0 | 0 | 0 | 8.8 | 0 | 0.02 | 0 | 0.34 | 0 | 0 |
| Psychrobacter | 0 | 0 | 0 | 0.01 | 0 | 0 | 1.19 | 6.51 | 0 | 0.02 |
| Flavobacterium | 0 | 0 | 0 | 0 | 0 | 0 | 6.89 | 9.04 | 0 | 0 |
| Clostridium | 0 | 0 | 0 | 0 | 0 | 0 | 8.61 | 0 | 0.12 | 0 |
| Others | 0.6 | 0.38 | 1.86 | 1.01 | 1.73 | 0.34 | 1.02 | 3.11 | 2.15 | 0.56 |

**S9 Table.** Correlation matrix showing pairwise Pearson’s r correlations between genera (bottom) and their associated significance (top).

|  |  |  |  |  |  |  |  |  |  |  |  |  |  |
| --- | --- | --- | --- | --- | --- | --- | --- | --- | --- | --- | --- | --- | --- |
| Genera | *Francisella* | *Acinetobacter* | *Bacillus* | *Corynebacterium* | *Escherichia* | *Flavobacterium* | *Lysinibacillus* | *Moraxella* | *Muribaculum* | *Pseudomonas* | *Psychrobacter* | *Staphylococcus* | *Uruburuella* |
| *Francisella* |  | 0.028888 | 0.2016 | 0.01161 | 0.042961 | 0.3991 | 0.39019 | 0.21176 | 0.62658 | 0.069064 | 0.82388 | 0.13323 | 0.61401 |
| *Acinetobacter* | -0.49 |  | 0.051909 | 0.90693 | 0.91299 | 0.96279 | 0.79911 | 0.76339 | 0.96784 | 0.073076 | 0.54225 | 0.95475 | 0.79837 |
| *Bacillus* | -0.3 | 0.44 |  | 0.47827 | 0.56815 | 0.60233 | 0.0088522 | 0.58148 | 0.61607 | 0.89166 | 0.57541 | 0.49103 | 0.4529 |
| *Corynebacterium* | -0.55 | -0.03 | -0.17 |  | 0.92492 | 0.5946 | 0.98558 | 0.090979 | 0.44631 | 0.93551 | 0.36876 | 0.011999 | 0.40722 |
| *Escherichia* | -0.46 | -0.03 | -0.14 | -0.02 |  | 0.13692 | 0.50345 | 0.52328 | 0.17241 | 0.037759 | 0.76825 | 0.85305 | 0.47266 |
| *Flavobacterium* | -0.2 | 0.01 | -0.12 | -0.13 | 0.34 |  | 0.70582 | 0.54796 | 0.46223 | 0.38783 | 0.33889 | 0.73006 | 0.55458 |
| *Lysinibacillus* | -0.2 | 0.06 | 0.57 | 0 | -0.16 | -0.09 |  | 0.86654 | 0.789 | 0.75434 | 0.81548 | 0.9468 | 0.49989 |
| *Moraxella* | -0.29 | 0.07 | 0.13 | 0.39 | -0.15 | -0.14 | 0.04 |  | 0.98666 | 0.38345 | 0.62304 | 0.65708 | 0.016011 |
| *Muribaculum* | 0.12 | 0.01 | -0.12 | 0.18 | -0.32 | -0.17 | -0.06 | 0 |  | 0.42652 | 0.43271 | 0.34805 | 0.84366 |
| *Pseudomonas* | -0.41 | 0.41 | 0.03 | -0.02 | 0.47 | 0.2 | 0.07 | 0.21 | -0.19 |  | 0.018532 | 0.95822 | 0.67982 |
| *Psychrobacter* | 0.05 | 0.14 | -0.13 | -0.21 | -0.07 | 0.23 | -0.06 | -0.12 | -0.19 | 0.52 |  | 0.72568 | 0.64318 |
| *Staphylococcus* | -0.35 | -0.01 | -0.16 | 0.55 | 0.04 | -0.08 | -0.02 | 0.11 | -0.22 | 0.01 | -0.08 |  | 0.82718 |
| *Uruburuella* | -0.12 | 0.06 | 0.18 | 0.2 | -0.17 | -0.14 | 0.16 | 0.53 | 0.05 | 0.1 | -0.11 | 0.05 |  |
